# Low Risk of Thromboembolic Complications After Fast-Track Abdominal Surgery With Thrombosis-Prophylaxis Only During Hospital Stay

**DOI:** 10.4021/gr320e

**Published:** 2011-05-20

**Authors:** Arne Christian Mohn, Jon Egge, Ola Rokke

**Affiliations:** a Department of Surgery, Haugesund Hospital, Haugesund, Norway; b Department of Radiology, Haugesund Hospital, Haugesund, Norway; c Department of Surgery, Akershus University Hospital, and Faculty of Medicine, University of Oslo, Oslo, Norway

**Keywords:** Thrombosis, Embolus, ERAS, Accelerated recovery, Fast-track surgery, Abdominal surgery, Cancer

## Abstract

**Background:**

Subcutaneous low molecular weight heparin (LMWH) reduces the risk of thromboembolic complications after abdominal surgery. With enhanced recovery after surgery (ERAS), median hospital stay after abdominal surgery may be as short as 3 - 4 days. The aim of our study was to investigate whether thrombosis prophylaxis during the short hospital stay was sufficient to maintain a low frequency of thromboembolic complications.

**Methods:**

Ninety-eight patients, median age 67 years, were enrolled in a prospective two-center observational study of colorectal resections following the ERAS principles. Seventy-seven patients (78.6%) were resected for colonic cancer, the rest for benign colonic diseases. Fifty percent of the patients were discharged from hospital within three days after surgery. Follow-up examinations took place at 8 and 30 days after surgery with clinical examination for thromboembolism. The patients enrolled at one of the centers were also scheduled for a routine venography at day 8. Seventeen of these were evaluated.

**Results:**

Clinical follow-up at day 8 of 72 patients (73.5%) revealed no venous thromboembolism (VTE), and the 17 venograms did not show any thromboses. Clinical follow-up at day 30 of 74 patients (75.5%) showed no deep venous thrombosis (DVT), whereas pulmonary embolus (PE) was suspected and verified in one patient (1.3%) with pulmonary metastases and pneumonia.

**Conclusions:**

Prophylaxis until full mobilization seems to be sufficient following major surgery in patients treated with the principles of ERAS who remain in hospital for 3 - 4 days.

## Introduction

Patients undergoing major surgery are at risk of developing deep venous thrombosis (DVT) or pulmonary embolus (PE). Without proper prophylaxis, hip prosthesis surgery and abdominal cancer surgery have a 70% and 30% risk of venous thromboembolism (VTE) respectively [1, 2]. The use of pre- and postoperative low molecular weight heparin (LMWH) has reduced the incidence of symptomatic VTE to around 3%, with an incidence of fatal pulmonary embolus of 0.2 - 0.7% [3]. There is, however, disagreement about the optimal duration of heparin-prophylaxis after surgery. Some authors argue for continued prophylaxis for four to six weeks after surgery, and have shown a reduction of VTE of 50% [4]. Most of these VTEs were asymptomatic, however, and the extensive prophylaxis does not eliminate fatal PE. Others argue for prophylaxis until full mobilization only [1], as has been the procedure in our department with traditional recovery principles.

In modern surgical departments there is a drive towards shorter hospital stay and more day care surgery. With the enhanced recovery after surgery (ERAS) principles, about 50% of patients may be fully mobilized and discharged from hospital within three days after colorectal resections [5]. In reports on ERAS-studies, thromboprophylaxis is given only during the hospital stay, without any increase in thromboembolic complications [5]. This may be due to the elements of ERAS, with food and drinks allowed until surgery, aggressive postoperative mobilization and the use of epidural analgesia, which may compensate for the reduced duration of thromboprophylaxis and the hypercoagulable state in cancer patients [6]. However, no systematic follow-up with regard to VTE has been performed so far in this patient group.

The aim of the present study was to survey the incidence of VTE after colorectal surgery using the ERAS principles, with thromboprophylaxis restricted to the hospital stay.

## Materials and Methods

Between October 2000 and April 2003, 103 consecutive patients scheduled for elective, open colorectal surgery without stoma were included in a prospective, non-randomized study of ERAS at Haukeland University Hospital (n = 71) and Haugesund Hospital (n = 27). Patients above 18 years, who gave written informed consent, were included. The exclusion criteria were pregnancy, patients with cognitive disorders that made it difficult for the patients to comply with the protocol, and changes in planned surgery from colonic resection to major upper abdomen surgery or stoma surgery. In this study we used the perioperative principles recommended by Kehlet et al. in order to enhance the patients’ recovery after surgery and reduce hospital stay [5]. One week before surgery the patients and relatives were informed by the surgeon and study nurse about the study program. The day of discharge was planned. On the day before surgery, the patients were allowed a normal diet with a supplement of protein drinks. A preoperative enema was given. Preoperative blood-samples were drawn from the patients treated at Haugesund Hospital and planned for ascending venography to investigate any disposition for thrombophilia [7, 8], and analyses of Protein S and C, APC-resistance, anti-cardiolipin antibodies, lupus anticoagulants and prothrombin were performed. The ethical committee did not allow more patients to be included in this part of the study. To reduce the surgical stress response and postoperative pain and nausea, we modified the anesthesia regime, including peri- and postoperative restriction of intravenous fluids, epidural analgesia at the level of TH 7 - 10 and total intravenous anesthesia (propofol, remifentanil og vekuron). A supplement of BIS (bispectral index) recordings was performed in Haugesund. Odansetron was not given perioperatively. The patients were mobilized out of bed postoperatively for two hours on the day of surgery. On the first postoperative day, the patients spent eight hours out of bed, with free access to food and fluids. Intravenous fluids and opiate administration were restricted. The urine catheter was removed on the first postoperative day and the epidural catheter on the second. Thromboprophylaxis was given in full dose enoxaparin/dalteparin (40 mg/5000 IE) the evening before surgery and half dose the evening after surgery. Prophylaxis was continued with full dose every evening during the hospital stay until discharge. After discharge the patients were encouraged to maintain physical activity. To stimulate and verify active mobilization, the patients were asked to fill out daily questionnaires one week after surgery, and then weekly until 30 days postoperatively. A clinical follow-up examination was performed 8 - 10 days and 30 days after surgery. The 27 patients treated at Haugesund Hospital were scheduled for venography to disclose deep venous thrombosis eight days after surgery. Venography was done by bilateral ascending venography according to Rabinov and Paulin [9], using between 50 - 150 ml iodixanol (iodixanol 270 mg/ml) in each leg to obtain images of the vein system. All venograms were examined by one radiologist and checked by a senior radiologist specializing in venograms. The criterion for a diagnosis was a constant defect seen on at least two images that were not filling defects. All analyses were on the basis of intention to treat. The results are given in the tables as median values (range). Since the period that the patients were given thromboprophylaxis before leaving the hospital was not consistent, the length of thromboprophylaxis is given in “hospital stay + one day or less”.

The study was approved by the Norwegian West Regional Ethical Committee.

## Results

Of the 103 patients included in the study, five patients were excluded because of extensive upper abdominal surgery, giving a study group of 98 patients. Characteristics of the patients and surgery are listed in Table 1. A total of 72 patients (73.5%) attended the first check-up 8 - 10 days after surgery (14 patients were still in hospital, 5 patients had been readmitted to hospital, 6 did not show up and one had died in hospital). A total of 74 patients (75.5%) attended the check-up 30 days after surgery (16 did not attend because of complications, 8 others dropped out). Complications occurred in 31 patients (31.6%). Median hospital stay was three days. Details of complications and hospital stay are given in Table 2. A total of 49 (50%) of the 98 patients included in the study were discharged from the hospital within three days, and thus received thromboprophylaxis for four days or less. The duration of thromboprophylaxis is shown in Table 3. Screening bilateral venography was planned for the 8th day after surgery for the 27 patients treated at Haugesund Hospital. However, three patients were too weak to participate and five dropped out. Venography was performed in 19 patients. Two had incomplete venograms, leaving venograms from 17 patients (63%) for further examination. Three of the remaining venograms were supplied by ultrasound scanning. Ten of these 17 patients were cancer patients, and one patient had a previous history of DVT. One patient had an old post-thrombotic finding. There were no complications after the venographies. There was no clinical VTE during their hospital stay and no asymptomatic thrombosis was discovered in the venograms. The group investigated by venograms had characteristics comparable to the patients who did not have venograms, aside from a trend towards shorter duration of thromboprophylaxis. The group of patients receiving venograms received shorter thromboprophylaxis: 76.4% received thromboprophylaxis for four days or less compared to 44.4% of the patients without venograms. During the 30 days postoperatively, a patient who had metastases in the lungs and liver (discovered during surgery) developed pneumonia and a non-fatal, symptomatic lung embolus (verified by CT-scan). No other events of symptomatic VTE were discovered.

**Table 1:** Patient Characteristics and Type of Surgery

	All patients (n = 98)	Venography (n = 17)
Age (years)	67 (19 - 90)	71 (44 - 89)
Weight (kg)	67 (35 - 124)	66 (35 - 124)
Gender		
Male	40 (40.8%)	8 (47.1%)
Female	58 (59.2%)	9 (52.9%)
ASA-score		
ASA 1	6 (6.1%)	0
ASA 2	69 (70.4%)	13 (76.5%)
ASA 3	23 (23.5%)	4 (23.5%)
Indication for surgery		
Cancer	77 (78.6%)	11 (64.7%)
Benign disease	21 (21.4%)	6 (35.3%)
Type of surgery		
Right hemicolectomy	52 (53.1%)	10 (58.8%)
Left hemicolectomy + sigmoid resection	30 (30.6%)	2 (11.8%)
Subtotal colectomy	5 (5.1%)	0
Stoma closure	6 (6.1%)	2 (11.8%)
Low anterior resection	4 (4.1%)	3 (17.6%)
Enterography	1 (1.0%)	0

**Table 2:** Complications and Hospital Stay

	All patients (n = 98)	Venography (n = 17)
Complications	31 (31.6%)	4 (23.5%)
Prolonged nausea/vomiting	7 (7%)	1 (5.9%)
Urinary tract infection	6 (6%)	1 (5.9%)
Pneumonia	6 (6%)	0
Superficial wound infection	5 (5%)	1 (5.9%)
Intra-abdominal abscess/septicemia	6 (6%)	1 (5.9%)
Anastomotic leakage	8 (8%)	1 (5.9%)
Cardiac infarction	1 (1%)	0
Thromboembolic complications in hospital	0	0
Thromboembolic events after discharge	1 (1%)	0
Mortality	1 (1%)	0
Hospital stay	3 (2 - 40)	3 (3 - 6)
Readmission	15 (15.3%)	3 (17.6%)

**Table 3:** Disposition for Thromboembolism and Thromboprophylaxis

	All patients (n = 98)	Venography (n = 17)
Previous history of DVT (n)	-	2
Thromboembolic disposition (n)	-	7 (41.2%)
AT-III deficiency	-	1 (5.9%)
Protein S deficiency	-	0
Protein C deficiency	-	0
Factor V Leiden mutation	-	1 (5.9%)
Cardiolipin antibodies	-	2 (11.8%)
Lupus anticoagulants	-	4 (23.5%)
Prothrombin mutation	-	0
Days with LMWH		
4 days or less	49 (50%)	13 (76.4%)
5 days	14 (14.3%)	2 (11.8%)
6 days	14 (14.3%)	0
7 days	7 (7.1%)	2 (11.8%)
> 7 days	13 (13.3%)	0
DVT by venography	-	0
DVT by clinical exam day 8	0	0
DVT by clinical exam day 30	0	0
PE during the first 30 days	1	0

The results of follow-up with regard to VTE, as well as the results of blood screening for thrombophilia, are shown in Table 3. Six patients (four venograms) were slightly positive on thromboembolic dispositions, but were probably of no significance. Two patients (none with venograms) were slightly positive on two different thromboembolic dispositions and may have some clinical relevance, one of them having had an earlier DVT episode. Four patients (three venograms) were positive on thromboembolic dispositions (two antithrombin II positive, one Factor V Leiden positive and one strongly positive IgM cardiolipin antibody and moderate positive lupus anticoagulantia), which strongly suggested clinical relevance. Two other patients had a history of VTE without thromboembolic dispositions.

## Discussion

Thromboembolic prophylaxis is normally terminated when the patients are mobilized and discharged from hospital. When following the principles of ERAS, hospital stay seems to be significantly shorter than after traditional recovery. This may be a concern with regard to thromboembolic complications. In the present study on ERAS in colorectal surgery, we were able to reproduce the results of Kehlet et al. [5], as 50% of our patients were discharged from hospital within three days, and received thromboprophylaxis (LMWH) for four days or less. This is considerably shorter than the normal length of thromboprophylaxis during traditional recovery of 7 - 14 days [1, 3, 8, 10, 11]. In spite of this, the incidence of thromboembolic complications at follow-up at 10 and 30 days after the operation was low in the present study. No deep venous thrombosis was detected by clinical examination at follow-up (0/98), and no asymptomatic thrombosis was detected on venograms of a cohort of 17 patients at day 8, even though some had thromboembolic dispositions and earlier VTE episodes. One pulmonary embolus (1%) was detected in a patient with pulmonary metastases and pneumonia. This incidence is not unfavorable compared to reported incidences of VTE following traditional recovery, where prophylaxis with LMWH gives an incidence of symptomatic and asymptomatic VTE of around 10% [12, 13] after colorectal surgery. Cancer patients seem to have a somewhat higher degree of events, at around 15% [10, 12, 13], with fatal pulmonary emboli (PE) occurring in 0.2 - 0.9% [1, 10, 13]. While medical illnesses such as cancer (hypercoagulability), obesity, previous VTE and thrombophilia may increase the incidence [8, 14, 15], perioperative care involving early mobilization or mechanical prophylaxis and a focus on fluid status, transfusion practice and regional anesthesia, may reduce VTE [1, 10, 16]. In our study, early mobilization based on the recommendations of Kehlet [5], restrictive perioperative intravenous fluid treatment, normal feeding intake on day one with epidural anesthesia for two days, early discharge from hospital, surveillance of the patients’ activity at home and early follow-up, may contribute to our favorable results.

In the ENOXACAN II study [4] the overall incidence of VTE was lower (12%) than previous studies such as the ENOXACAN I study (15%) [4, 12]. One explanation may be the increased focus on early mobilization and early discharge from hospital [4]. Another observational study by Hidalgo et al., of patients mobilized early after repair of hernias in the abdominal wall, found a median hospital stay of 2.5 days, and only two cases (1.2%) of VTE [17], which may be explained in the same way. Our median duration of prophylaxis was no more than four days, which is less than normally recommended, but the treatment lasted until full mobilization. The full level of mobilization continued after discharge as verified by daily and weekly questionnaires. Kehlet et al. [5] did not report a higher incidence of VTE even though the median duration of prophylaxis was no more than three days. Although they did not comment on it, the incidence was shown to be lower in a review article by Kehlet and Wilmore [18]. As in Hidalgo et al., it seems that the shorter treatment time does not interfere with the incidence of VTE. Even more interesting is the incidence of VTE in a study by Gonzales et al. [19], in which morbidly obese patients were treated without heparin, using only pneumatic compression hoses. Gastric bypass in morbidly obese patients is normally considered as high-risk surgery. Only one of 380 patients had a clinically verified DVT (0.26%) on duplex ultrasonography. The reason may be that early mobilization and/or pneumatic compression (mechanical prophylaxis) with regional analgesia and continued mobilization compensate for shorter treatment time, major surgery and cancer hypercoagulability. In that sense treatment for four to six weeks after surgery would be over-treatment, especially since the low level of fatal PE is not eliminated after prolonged prophylaxis.

Ascending venography has been the gold standard for detecting venous thrombus in clinical studies. However there are limitations to this method of examination. The technique is invasive with possible, but rare, complications. Incomplete venograms and discomfort for the patients cause patient exclusion rates of between 25% and 40% [1, 4, 13, 20, 21]. In our study, 37% of the patients scheduled for venography were excluded for these reasons. Venography is also not repeatable and has inter-observer variability. Finally, there are still questions about the significance of small, distal thrombi, especially the asymptomatic ones. Therefore, the standard is changing towards venous Doppler ultrasonography, which can be repeated, is non-invasive and is highly sensitive to symptomatic thrombi of the lower extremities and asymptomatic, proximal DVT [1, 2, 22], even though ultrasound has a lower sensitivity than venography [10, 13]. We found no DVTs by venography in 17 patients eight days after surgery. The patient cohort in which venography was performed also seems to be representative compared to the whole study population. Patients in the venogram-cohort were comparable with regard to age, weight, ASA-score, disease, surgical procedure, complications and duration of thromboprophylaxis. Based on the incidence of VTE in previous studies, we would expect at least two asymptomatic DVT in our small cohort [13], but neither the three patients with clear evidence of thrombophilia, including one heterozygote for Factor V Leiden, nor those who previously had a VTE, showed any signs of thromboemboli. However, we would not expect clinical findings of VTE in our small study since the rate that patients receive medical prophylaxis is only 0.2% when a clinical examination is used [10]. Even though there are case studies of distal thrombi propagating to cause embolies [10, 23] the proximal thrombi detected by ultrasound are mainly due to the emboli [1].

The usual duration of thromboprophylaxis is 7 - 14 days, using traditional recovery. Some authors recommend prolonged prophylaxis for one month, and have reported a reduction of relative incidence of DVT by 47 - 60% [4, 21, 24] on venographically detected DVT. Prophylaxis for 7 - 10 days after knee and hip arthroplasty had a 3.5 - 4% incidence of symptomatic VTE three months after surgery [8, 14]. Extended prophylaxis reduced this incidence to 1.3% [3, 25]. This strategy, however, does not eliminate the occurrence of fatal PE, and the reduction of symptomatic DVT was statistically significant in only two out of nine randomized controlled trials [3].

Some authors recommend long-duration medical prophylaxis (4 - 6 weeks) in patients receiving major surgery to reduce VTE that is mostly detected by routine testing that is no longer used (e.g., venography). There is a clear reduction of asymptomatic DVT and a tendency for a reduction of symptomatic DVT, but PE is not eliminated. Others promote medical and mechanical prophylaxis until full mobilization with a VTE rate of 0.2% on clinical examination.

Our study supports the latter authors. Until there are large studies on the cost-benefits, the discussion will not come to an end.

### Conclusion

In the present study we demonstrated a low incidence of thromboembolic complications after short-term LMWH-prophylaxis during ERAS. The study supports the reports and studies that argue for treatment until full mobilization rather than prolonged prophylaxis.
